# Antenatal care trial interventions: a systematic scoping review and taxonomy development of care models

**DOI:** 10.1186/s12884-016-1186-3

**Published:** 2017-01-06

**Authors:** Andrew Symon, Jan Pringle, Soo Downe, Vanora Hundley, Elaine Lee, Fiona Lynn, Alison McFadden, Jenny McNeill, Mary J Renfrew, Mary Ross-Davie, Edwin van Teijlingen, Heather Whitford, Fiona Alderdice

**Affiliations:** 1 Mother & Infant Research Unit, University of Dundee, DD1 4HJ Dundee, UK; 2 School of Nursing & Health Sciences, University of Dundee, DD1 4HJ Dundee, UK; 3 School of Health, Brook Building, University of Central Lancashire, Preston, PR1 2HE UK; 4 Centre for Midwifery, Maternal & Perinatal Health, Faculty of Health & Social Sciences, Bournemouth University, BU1 3LH Poole, UK; 5 School of Nursing & Midwifery, Queens University, Belfast, BT9 7BL UK; 6 Maternal & Child Health, NHS Education for Scotland, Edinburgh, EH3 9DN UK

**Keywords:** Pregnancy, Prenatal care, Antenatal care, Model of care, Health services research, Randomised controlled trial, Systematic review, Pregnancy outcome, Taxonomy

## Abstract

**Background:**

Antenatal care models vary widely around the world, reflecting local contexts, drivers and resources. Randomised controlled trials (RCTs) have tested the impact of multi-component antenatal care interventions on service delivery and outcomes in many countries since the 1980s. Some have applied entirely new schemes, while others have modified existing care delivery approaches. Systematic reviews (SRs) indicate that some specific antenatal interventions are more effective than others; however the causal mechanisms leading to better outcomes are poorly understood, limiting implementation and future research. As a first step in identifying what might be making the difference we conducted a scoping review of interventions tested in RCTs in order to establish a taxonomy of antenatal care models.

**Methods:**

A protocol-driven systematic search was undertaken of databases for RCTs and SRs reporting antenatal care interventions. Results were unrestricted by time or locality, but limited to English language. Key characteristics of both experimental and control interventions in the included trials were mapped using SPIO (Study design; Population; Intervention; Outcomes) criteria and the intervention and principal outcome measures were described. Commonalities and differences between the components that were being tested in each study were identified by consensus, resulting in a comprehensive description of emergent models for antenatal care interventions.

**Results:**

Of 13,050 articles retrieved, we identified 153 eligible articles including 130 RCTs in 34 countries. The interventions tested in these trials varied from the number of visits to the location of care provision, and from the content of care to the professional/lay group providing that care. In most studies neither intervention nor control arm was well described. Our analysis of the identified trials of antenatal care interventions produced the following taxonomy: *Universal provision model* (for all women irrespective of health state or complications); *Restricted ‘lower-risk’-based provision model* (midwifery-led or reduced/flexible visit approach for healthy women); *Augmented provision model* (antenatal care as in Universal provision above but augmented by clinical, educational or behavioural intervention); *Targeted ‘higher-risk’-based provision model* (for woman with defined clinical or socio-demographic risk factors). The first category was most commonly tested in low-income countries (i.e. resource-poor settings), particularly in Asia. The other categories were tested around the world. The trials included a range of care providers, including midwives, nurses, doctors, and lay workers.

**Conclusions:**

Interventions can be defined and described in many ways. The intended antenatal care population group proved the simplest and most clinically relevant way of distinguishing trials which might otherwise be categorised together. Since our review excluded non-trial interventions, the taxonomy does not represent antenatal care provision worldwide. It offers a stable and reproducible approach to describing the purpose and content of models of antenatal care which have been tested in a trial. It highlights a lack of reported detail of trial interventions and usual care processes. It provides a baseline for future work to examine and test the salient characteristics of the most effective models, and could also help decision-makers and service planners in planning implementation.

**Electronic supplementary material:**

The online version of this article (doi:10.1186/s12884-016-1186-3) contains supplementary material, which is available to authorized users.

## Background

In an attempt to establish an evidence base for improving pregnancy outcomes over recent decades, a range of antenatal care interventions, reflecting local contexts, political drivers and financial considerations, have been the subject of randomised controlled trials (RCTs). These RCTs have ranged from applying an intervention that is an entirely new form of antenatal care provision (usually where existing provision was scanty or there was a desire to introduce a fundamentally different approach), to modifying the care delivery when existing services were already sophisticated, e.g. additional clinics for specific groups of women.

Within many high-income countries the increasing acceptance of women’s rights regarding choice and autonomy in maternity care has led to a more woman-centred approach to antenatal care. For example, United Kingdom (UK) maternity care has been driven by policies to empower service users [1], with evaluations of women’s experiences [2–4]. Similar drivers have been noted in Australia [5] and New Zealand [6]. Several RCTs have reflected the increasing focus on woman-centred care by incorporating continuity of care elements [7, 8], often involving team or caseload midwifery. Team midwifery is defined as a group of midwives providing care and taking shared responsibility for women from the antenatal period, through labour and on to postnatal care [9]. In caseloading, a midwife is responsible for the continuum of care throughout pregnancy, birth and the postnatal period for a small identified number of women [10]. The impetus to provide high standards in maternity care is not limited to high-income countries, as intervention trials have occurred around the world. *The Lancet* Series on Midwifery produced the global evidence-based Quality Maternal and Newborn Care (QMNC) Framework [11] which emphasises this desire to provide all women with high quality maternity care. Such care has long been recognised as providing a sound foundation for the health of future generations [12], although the resources needed to provide such care are not evenly distributed round the world. The World Health Organisation (WHO) antenatal care standards have just been updated [13], and now recommend eight visits. At the time of our review the recommendation was for a minimum of four visits to a skilled birth attendant with specific activities to be undertaken for each visit.

Cochrane reviews have found that adverse outcomes, including preterm birth, could be reduced by ‘midwife-led continuity of care’ provision [7, 8], while a matched cohort study of women accessing an independent midwife (IM) in the UK and women receiving ‘standard’ National Health Service (NHS) care found a significantly reduced preterm birth rate in the IM group [14]. Independent midwives provide care on a contractual basis outside of routine state-funded provision. A review [15] of ‘non-standard’ maternity care models in the United States (US), UK and Australia found an association with increased attendance for antenatal care, fewer preterm births, and increased breastfeeding initiation. Although concerns have been expressed about methodological limitations of its evaluations, the CenteringPregnancy™ (CP) group-based model of antenatal care and education has been evaluated positively, demonstrating both clinical benefits and improved social outcomes [16]. The question of the cost-effectiveness of different models of care is also important [17], especially in relation to outcomes with long-term sequelae such as preterm birth [18], although some economic analyses do not distinguish birth centre and continuity models. A Cochrane review [19] of group-based antenatal care found no improvements in clinical outcomes, although the four included trials were quite heterogeneous.

In order to clarify the various interventions that have been attempted and achieved, and to categorise similar approaches so that a taxonomy of models can be produced, we initiated a systematic scoping review of RCTs and systematic reviews (SRs) of RCTs. In this article we use the term ‘intervention’ when referring to particular trials, and ‘model’ when referring to the resulting taxonomy. Because the term ‘model’ is frequently used when referring to an individual approach which might be the subject of a trial, we also used this as a search term (see below). The review was started in 2014 under the auspices of the multi-disciplinary and multi-institution McTempo research collaboration (Models of Care: The Effects on Maternal & Perinatal Outcomes). This research is timely because of the recent focus on global midwifery and what good quality maternal and newborn care should encompass [11]. The overall aim of the McTempo research collaboration is to examine the association between and possible mechanisms within different models of antenatal care and a range of clinical, psychosocial and organisational outcomes. This scoping review was undertaken as an initial step towards this aim.

Taxonomies (Greek: ‘arrangement method’) were historically used as classification systems within biology, but have also been used in other disciplines [20]. As a basis for future work in this area, the specific aim of this study was to develop a taxonomy of antenatal care interventions that have been tested in RCTs.

## Methods

A study protocol was developed and search strategies were implemented accordingly. Primary searches were conducted by the York Health Economics Consortium during July-August 2014. The search architecture comprised three concepts: antenatal care; models linked to carers – non-midwife specific; models linked to midwives. These concepts were combined as follows: (Antenatal care AND Models linked to carer – non-midwife specific) OR Models linked to midwives. The strategy also included additional focused stand-alone searches on potentially relevant antenatal model terms (‘antenatal’ and synonyms; model; group/individual/team/caseload/shared/integrated/multidisciplinary). Table 1 shows inclusion and exclusion criteria, and the databases searched. The search was restricted to SRs and RCTs, without a date restriction.

**Table 1:** Databases searched, and inclusion and exclusion criteria

Database/information source	Inclusion criteria	Exclusion criteria
British Nursing Index Cochrane Database of Systematic Reviews Cochrane Central Register of Controlled Trials (CENTRAL) CINAHL Plus ClinicalTrials.gov Science Citation Index Expanded (SCI-EXPANDED) Social Sciences Citation Index (SSCI) Conference Proceedings Citation Index- Science (CPCI-S) Conference Proceedings Citation Index- Social Science & Humanities (CPCI-SSH) Database of Abstracts of Reviews of Effects (DARE) Econlit Embase Health Technology Assessment (HTA) Database International Clinical Trials Registry Platform (ICTRP) Maternity and Infant Care MEDLINE In-Process & Other Non-Indexed Citations and MEDLINE POPLINE PsycINFO NHS Economic Evaluation Database (EED) HEED: Health Economic Evaluations Database	• *Study design*: RCTs and reviews of RCTs, with meta-analysis • *Population*: pregnant women receiving antenatal care, +/- family or people delivering such care • *Intervention*: any model offering a defined package of antenatal care, which might be delivered by professional staff or peer/non-professional supporter • *Outcome(s)*: maternal/infant perinatal outcomes; maternal psychosocial outcomes; organisational outcomes, including economic evaluations; maternal health behaviour outcomes	• Reviews of mixed methods studies, where the results of any RCTs were not clearly identifiable from other results and/or they did not contain a meta-analysis (and therefore did not add to original papers) • Interventions only offered during labour and/or the postnatal period • Stated outcomes were not relevant to pregnancy, birth or the period following birth (further defined as up to 2 years following birth) • The main area of interest or outcome related specifically to child abuse • An English language version was not available (due to translation and resource limitations)

The titles and abstracts of the articles obtained through this process were screened independently by paired members of the team; those identified as suitable for full text review were then screened using SPIO criteria (Study design, Population, Intervention, Outcome). For this purpose of producing a taxonomy of models we did not perform a formal quality assessment of all the identified papers. We have done so for specific sub-groups in order to achieve other goals within the project team’s remit [21]. We made the primary study the main focus of this taxonomy. We have distinguished primary and sibling papers reporting empirical studies, as well as the systematic reviews which included such studies.

Scrutiny and evaluation of the intervention was carried out by the McTempo team who classified the relevant trials according to the target group, type of intervention offered, who was involved in its delivery, how it was organised, and where these interventions typically took place. As this was a scoping review this was an iterative process. Commonalities and differences were identified through group discussion, resulting in eventual consensus. While we broadly followed the Arksey and O’Malley [22] methodological framework for scoping reviews we did not adjust our inclusion and exclusion criteria *post hoc*; in addition, we restricted ourselves to higher levels of research evidence, namely RCTs and systematic reviews of RCTs.

## Results

**Fig. 1:**
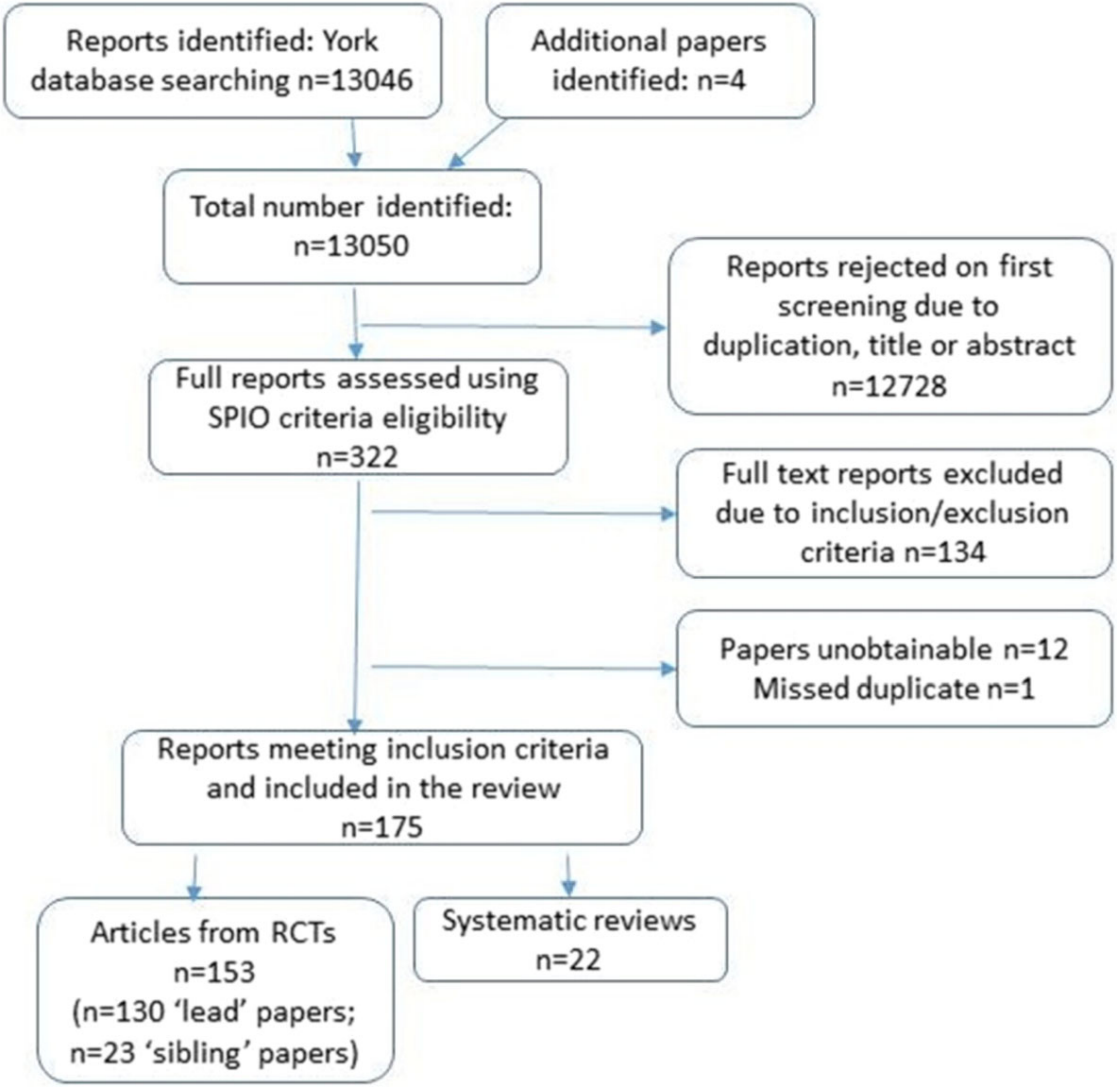
PRISMA flow chart

The searches found 13,046 papers and four papers were subsequently identified from reference lists. Following screening of titles and abstracts, 322 papers were identified for full text screening. Of those, 134 papers were excluded at this stage, and 12 papers were unobtainable (largely due to length of time since publication). There was one missed duplicate identified at the full text stage. This process is detailed in the PRISMA flow chart in Fig. 1.

As Fig. 1 indicates, 153 articles reporting 130 interventions involving antenatal care were identified for full review, as were 22 associated SRs. These papers related to clinical care and/or organisational delivery, and could contain educational or lifestyle elements. All interventions covered the antenatal period; some also included intrapartum and postnatal care (Table 2). While some papers provided information about the trial’s intervention arm, this was often scanty (cf. [23]). Similarly, the control arms were rarely described in any detail. Despite relevant authors being contacted, 12 papers were unobtainable, ten dating from 1985 to 98.

**Table 2:** Taxonomy of Experimental Antenatal Interventions tested within an RCT

Care models	Type of care/intervention	Personnel	Organisation	Location
Universal provision model – no restrictions on eligibility	Clustered community-focussed models	TBAs; skilled birth attendants (midwives, nurses, physicians, ‘lady health workers’ etc.), health committees; community workers; health facilitators/educators/trainers; volunteers	Task-based; participatory women’s groups; learning and action cycle; mostly group-based within community settings; Lack of continuity of carer	Home; community setting; health centre; commune
	Reduced/flexible visits	Midwives, GPs (general practitioners/family physicians), obstetricians	Task-based; individual focus; lack of continuity	Hospital; urban community; clinic
	Hospital-based group models	Midwives		Antenatal clinic
	Midwifery-led models (some allowed mixed risk)	Midwives; obstetric nurses; rural practice team	Midwifery-led; Continuity of care/carer; sometimes task-based.	Antenatal clinic; teaching hospital, rural clinic
Restricted ‘lower risk’-based model – eligibility limited to (‘low risk’ women)	Midwifery-led models	Midwives, with medical support as required	Midwife-led; Woman-centred; Continuity of care/carer	Home/community setting and/or hospital/institutional setting; birth centre.
	Reduced/flexible visits	Midwives, obstetricians, GPs (general practitioners/family physicians); OB-GYNs (obstetrician-gynecologists); certified nurse-midwives.	Task-based; individual focus; lack of continuity; some flexibility.	Hospital clinic; GP surgery; birth centre.
Augmented provision model – no restrictions on eligibility but with additional care given	Supplementary antenatal care or educational input	Psychotherapists; health care professionals (physicians; nurses; midwives); home visitors; community health workers; nurse-midwives	Group or individual focus; structured health education; continuity of care; family-focussed; case-note holding	Health centre; clinic; hospital; home
	Behavioural or lifestyle intervention, including effect of exercise on gestational weight gain, pregnancy outcomes, breast feeding, relaxation etc.	Dieticians; fitness instructors; obstetricians; ‘interventionist’ physiotherapists; midwives	Individual counselling; exercise training; dietary advice, weight management; hypnosis; task-based	Clinic; community; hospital; home
Targeted ‘higher risk’-based model - for specific groups	Specific care for women with identified Clinical/Psychosocial/Socio-demographic risk factors:	Physicians and/or midwives/nurses	Task-based or woman-centred Continuity of care/carer or no continuity	Hospital/institutional setting

We acknowledge that classifying interventions is complex and open to debate, in part because of the language/terms used. For example, an intervention based on ‘continuity’ or ‘midwife-led’ care could also be classified as a group-based intervention; an augmented care approach could contain elements of continuity of care and also adopt a group-based approach, etc. Our rationale for distinguishing the interventions relates principally to their target population, but also takes into account other contextual factors. Inevitably, there is a degree of overlap between some of the interventions, and the distinctions we have drawn in order to produce this taxonomy are open to debate. There is some overlap in terms of service provision between the Universal provision and the Restricted ‘lower risk’-based models, for example. All the interventions were covered by a mix of public and/or private financing, with or without an insurance element. Following the initial classification of the studies, the results were reviewed by the McTempo team. Minor subsequent adjustments resulted in an agreed classification taxonomy (Table 2). We emphasise that our review concerned interventions which have been tested in trials; we did not review all maternity care models worldwide.

Our taxonomy identified four classifications of antenatal care models:

1. *Universal provision model*. In this model the full spectrum of antenatal care was available to all women. We have used the term ‘midwifery-led’ rather than ‘midwife-led’ because the care provided in some studies reported here, while constituting midwifery, involved non-midwives whose practice and scope was not covered by the standard international definition of a midwife [24].
2. *Restricted ‘lower risk’-based provision model*. This model had some components in common with the ‘Universal provision’, but eligibility was restricted to women considered to be ‘low risk’. The definition of ‘low risk’ varied across trials/settings. In the Discussion we consider the potential pitfalls of using the term ‘risk’ within this taxonomy.
3. *Augmented provision model*. These trials were not restrictive in terms of eligibility, but comprised standard antenatal care augmented by additional clinical or educational input, or specific behavioural interventions. A sub-set of this category included trials which featured lifestyle interventions where the primary outcome was not pregnancy-related but focussed on lifestyle issues (e.g. smoking, nutrition, exercise).
4. *Targeted ‘higher risk’-based provision model*. These trials included woman deemed to have ‘higher risk’ status (whether for clinical, psychosocial or socio-demographic reasons), and provided care tailored to that specific risk status.

A range of types of care went to make up each model. Tables 3, 4, 5 and 6 describe these categories’ population groups and the focus of the relevant interventions, and cite the primary paper from each trial. It indicates the country in which that trial took place, and if the trial had any sibling papers or was included in any associated systematic reviews. It also specifies each category’s reference list.

**Table 3:** Universal provision model (25 studies and 7 systematic reviews)

Category	Description	Universal provision: Principal paper from RCT First author; year; [country of study]; * indicates sibling paper(s) – see relevant reference list; (SR1 etc. if included in a related systematic review)	Systematic Reviews associated with this category
Community delivered interventions (*n* = 14 principal papers and 1 SR) (see Additional file 1 reference list T3-1)	Interventions were mainly delivered in poor rural areas and underserved communities, typically in Asian/South Asian settings	Azad 2010 [Bangladesh] (SR1,16) Bhutta 2011 [Pakistan] Colborn 2013 [Malawi] (SR1) Darmstadt 2010 [Bangladesh] Fottrell 2013 [Bangladesh] (SR1) Jokhio 2005 [Pakistan] Lewycka 2013 [Malawi] (SR1) Manandhar 2004 [Nepal] (SR1) Midhet 2010 [Pakistan] Miller 2012 [Pakistan] More 2012 [India] (SR1) Mullany 2007 [Nepal] Pasha 2013 [India, Pakistan, Kenya, Zambia, Guatemala, Argentina] Persson 2013 [Vietnam]	1 Prost et al. 2013
Midwifery-led interventions (*n* = 5 principal papers, and 3 SRs) (see Additional file 1 reference list T3-2)	Studies where the main focus was on the impact of antenatal care delivered by midwives, or a comparison of midwifery-led care with another mode/model of delivery	McLachlan BK 2000 [UK - England] (SR2,3) Rowley 1995 [Australia] (SR2,3,4) Tracy 2013 [Australia] Walker 2013 [Mexico] Wu 2011 [China]	2 Devane et al. 2010 3 Sandall et al. 2013 4 Waldenstrom et al. 1998
Reduced/flexible visit interventions (*n* = 4 principal papers and 1 SR) (see Additional file 1 reference list T3-3)	Models investigating whether reduced or flexible antenatal visits had an impact on maternal/infant outcomes	Clement 1996 [UK – England] Majoko 2007 [Zimbabwe] (SR7,9,10) Munjanja 1996 [Zimbabwe] (SR5,8,9,10) Villar 2001 [Argentina, Cuba, Saudi Arabia, Thailand] (SR2,5,7,9,10)	5 Carroli et al. 2001
Group-based antenatal care interventions (*n* = 2 principal papers, 1 sibling paper and 1 SR) (see Additional file 1 reference list T3-4)	Comparing group-based and individual antenatal care	Andersson 2013 [Sweden] (SR6) Jafari 2010 [Iran] * (SR6)	6 Homer et al. 2012 (see Additional file 1 reference list T3-4)
Multiple foci (*n* = 1 SR) (see Additional file 1 reference list T3-5)		N/A	7 Yakoob et al. 2009 – interventions that impact on stillbirth

**Table 4:** Restricted ‘lower risk’-based model (18 studies and 8 systematic reviews)

Category	Description	Restricted risk-based provision: Principal paper from RCT First author; year; [country of study]; * indicates sibling paper(s) – see relevant reference list; (SR1 etc. if included in a related systematic review)	Systematic Reviews associated with this category
Midwifery-led interventions (*n* = 12 principal papers, 8 ‘sibling’ papers and 5 SRs) (see Additional file 1 reference list T4-1)	Studies where the main focus was on the impact of antenatal care delivered by midwives, or a comparison of midwifery-led care with another mode/model of delivery	Begley 2011 [Ireland] (SR2,3) Biro 2000 [Australia] * (SR2,3) Flint 1989 [UK - England] (SR2,3,4) Giles 1992 [Australia] (SR8,9) Gu 2013 [China] Harvey 2002 [Canada] (SR2) Hicks 2003 [UK - England] (SR2,3) Homer 2001 [Australia] * (SR2,3,7) McLachlan HL 2012 [Australia] (SR3) Turnbull 1996 [UK - Scotland] *** (SR2,3,4,8,9) Waldenstrom 1994 [Sweden] ** Waldenstrom 2000 [Australia] * (SR2,3)	2 Devane et al. 2010 8 Khan-Neelofur et al. 1998 3 Sandall et al. 2013 – 9 Villar et al. 2007 4Waldenstrom et al. 1998
Reduced/flexible visit interventions (*n* = 6 principal papers, 1 sibling paper and 4 SRs) (see Additional file 1 reference list T4-2)	Models investigating whether reduced or flexible antenatal visits had an impact on maternal/infant outcomes	Henderson 2000 [UK – England] Jewell 2000 [UK – England] McDuffie 1996 [USA] * (SR5,8,9,10) Sikorski 1996 [UK – England] (SR5,8,9,10) Tucker 1996 [UK – Scotland] (SR8,9) Walker 1997 [USA] (SR5,8,9,10)	5 Carroli et al. 2001 10 Dowswell et al. 2010 8 Khan-Neelofur et al. 1998 9 Villar et al. 2007
Multiple foci (*n* = 1 SR) (see Additional file 1 reference list T4-3)		N/A	7 Yakoob et al. 2009 – interventions that impact on stillbirth

**Table 5:** Augmented provision model (20 studies and 6 systematic reviews)

Category	Description/details	Principal paper from RCT First author; year; [country of study]; (SR1 etc. if included in a related systematic review)	Systematic Reviews associated with this category
Additional care interventions (*n* = 13 principal papers and 1 SR) (see Additional file 1 reference list T5-1)	Studies where supplementary antenatal care or educational input was given to all pregnant women (i.e. not targeted because of perceived risk status)	Au 2006 [Canada] Bergstrom 2009 [Sweden] Ekstrom 2006 [Sweden] Ekhtiari 2014 [Iran] Elbourne 1987 [UK – England] (SR11) Hajian 2012 [Iran] Hemminki 2013 [China] Jennings 2010 [Benin] Leung 2012 [Hong Kong] Nsibande 2013 [South Africa] Nuraini 2005 [Indonesia] Svensson 2009 [Australia] Tough 2006 [Canada]	11 Brown & Smith 2004 – women carrying own notes 12 Dennis & Kingston 2008 – additional telephone support
Behavioural interventions (*n* = 7 principal papers and 4 SRs) (see Additional file 1 reference list T5-2)	These focussed on behavioural or lifestyle issues for all pregnant women, including effect of exercise on gestational weight gain, pregnancy outcomes, breast feeding, relaxation etc.	Asbee 2009 [USA] Barakat 2009 [Spain] (SR13,15) Barakat 2013 [Spain] (SR13) Phelan 2011 [USA] (SR15) Rakhshani 2010 [India] Stafne 2012 [Norway] (SR13) Werner 2013 [Denmark]	13 Domenjoz et al. 2014 – physical activity; 14 Ota et al. 2012 – dietary supplementation; 15 Ruifrok et al. 2014 – dietary and lifestyle interventions 16 Sibley et al. 2012 – the effect of TBA training on health behaviours;

**Table 6:** Targeted ‘higher risk’-based model (67 studies and 6 systematic reviews)

Category	Description/details	Principal paper from RCT First author; year; [country of study]; * indicates sibling paper(s) – see relevant reference list; (SR1 etc. if included in a related systematic review)	Systematic Reviews associated with this this category
‘Higher risk’ target groups (based on clinical/psychosocial indicators)			
Interventions for women with various or multiple risks (*n* = 9 principal papers, 3 sibling papers and 3 SRs) (see Additional file 1 reference list T6-1)	Generally involving augmented targeted care with a risk reduction focus [Higher risks not always well specified]	Brooten 2001 [USA] (SR20) Dawson 1989 [UK – Wales] * (SR20) Dawson 1999 [UK – Wales] (SR20) El Mohandes 2011 [USA] Kemp 2011 [Australia] * Klerman 2001 [USA] (SR20) Lee 2009 [USA] Turnbull 2004 [Australia] * (SR19) Villar 1992 [Argentina, Cuba, Mexico] (SR18,20)	17 Blondel & Breart 1992 - Home visiting for pregnancy complications 18 Blondel & Breart 1995 - Social or medical care for high risk women 19 Dowswell et al. 2009 - Care for women with complicated pregnancy (antenatal day care versus hospitalisation)
Interventions for women at risk of preterm birth or having a low birthweight (LBW) baby (*n* = 10 principal papers, 1 sibling paper and 1 SR) (see Additional file 1 reference list T6-2)	Interventions aimed at prevention of preterm birth or lbw baby	Bryce 1991 [Australia] (SR18) Depp 1993 [USA] Heins 1990 [USA] (SR20) Langer 1993 [Latin America] * Lutenbacher 2014 [USA] Mueller-Heubach 1989 [USA] Muender 2000 [USA] Norbeck 1996 [USA] (SR12,20) Oakley 1990 [UK – England] (SR12,18,20) Ross 1994 [USA]	20 Hodnett et al. 2010 - support for women at risk of LBW baby
Interventions for women who smoke (*n* = 9 principal papers and 1 sibling paper) (see Additional file 1 reference list T6-3)	Various approaches (individualised social support; group evening class; computer-delivered; telephone support; health practice population screening) aimed at encouraging smoking cessation	Bullock 2009 [USA] O’Connor 1992 [[Canada] Ondersma 2012 [USA] Parker 2007 [USA] Pbert 2004 [USA] Petersen 1992 [USA] Ruger 2009 [USA] Secker-Walker 1998 [USA] * Tappin 2000 [UK – Scotland]]	N/A
Interventions for women with anxiety or mental health issues (*n* = 9 principal papers) (see Additional file 1 reference list T6-4)	Studies involving additional support (e.g. home visits, relaxation classes, cognitive behavioural interventions)	Bastani 2006 [Iran] Brugha 2000 [UK - England] Guardino 2014 [USA] Ortiz Collado 2014 [Spain, France] Petrou 2006 [UK - England] Rahman 2008 [Pakistan] Richter 2012 [Germany] Saisto 2001 [Finland] Webster 2003 [Australia]	N/A
Interventions for overweight/obese women and/or women at risk of Gestational Diabetes Mellitus (*n* = 5 principal papers) (see Additional file 1 reference list T6-5)	Interventions to assist lifestyle behaviour change (e.g. exercise, telemedicine)	Harrison 2013 [Australia] Oostdam 2012 [Netherlands] (SR13) Perez Ferre 2010 [Spain] Poston 2013 [UK – Scotland & England] Quinlivan 2011 [Australia] (SR15)	N/A
Other ‘higher risk’ clinical/psychosocial target groups (see Additional file 1 Reference list T6-6)			
Interventions for women/babies at risk of abuse (*n* = 3 principal papers)	Interventions for those at risk of abuse	Barlow 2007 [UK – England] McIntosh 2009 [UK – England] Taft 2011 [Australia]	
Interventions for women with alcohol risk (*n* = 2 principal papers and 1 SR)	Brief alcohol counselling interventions for women identified as having a previous or current alcohol risk	O’Connor 2007 [USA] Osterman 2012 [USA]	21 Turnbull et al. 2012
Interventions for women with history of previous caesarean (*n* = 1 principal paper)	Antenatal education and support relating to vaginal birth.	Fraser 1997 [Canada, USA]	N/A
Interventions for women with HIV (*n* = 1 principal paper)	Telephone support intervention for pregnant women with HIV-positive status	Ross 2013 [Thailand]	N/A
Interventions for women with twin pregnancy (*n* = 1 principal paper and 1 sibling paper)	Preparation for twin birth programme	Sen 2006 [UK – England] *	N/A
‘Higher risk’ socio-demographic target groups (see Additional file 1 Reference list T6-7)			
Interventions for adolescent/younger age women (*n* = 8 principal papers and 2 sibling papers)	Additional support (e.g. home visits, education) to improve outcomes for ‘at risk’ group	Aracena 2009 [Chile] Barlow A 2006 [USA] Barlow A 2013 [USA] Barlow J 2007 [USA] Barnet 2002 [USA] Barnet 2007 [USA] Ford 2002 [USA] Ickovics 2007 [USA] ** (SR6)	22 Barlow J et al. 2011
Interventions for low income women (*n* = 6 principal papers and 5 sibling papers)	Community support interventions	Edwards 2013 [USA] McLachlan 1992 [USA] (SR20) Olds 1986 [USA] **** (SR18,20) Polley 2002 [USA] (SR15) Roman 2009 [USA] * Wen 2011 [Australia]	N/A
Interventions for women in the military/Military wives (*n* = 3 principal papers)	Interventions for military women or partners of military personnel to support adaptation to motherhood	Fausett 2014 [USA] Kennedy 2011 [USA] (SR6) Weis 2012 [USA]	N/A

### Universal provision model

Trials in this model fell into four main categories, with an additional ‘multiple focus’ category for a related systematic review. The interventions were community-based or midwifery-led, or involved reduced or flexible visits or group-based care. See reference lists T3-1 to T3-5 (Additional file 1).

#### Community delivered interventions

These 14 ‘community delivered’ interventions were mainly provided in low resource settings with limited existing antenatal service provision; there was one associated SR [25] which included one study not retrieved by our search. These interventions are a relatively recent innovation: the cited papers range from 2004 to 2013.

Interventions typically involved regular community group meetings for women of child-bearing age. Two interventions reported positive outcomes relating to the inclusion of husbands/partners in interventions [26, 27]. Across the other studies in this group, there were outcome commonalities of infant/maternal morbidity and mortality; indeed Prost et al.’s meta-analysis [25] found that such interventions have the potential to improve maternal and infant mortality, and reduce stillbirth rates in low resource settings.

#### Midwifery-led interventions

The earliest of these five studies was published in 1995 [28], with the most recent being the Tracy and Walker studies in 2013. As noted above, we have deliberately used the term ‘midwifery-led’, and in this we are drawing on some of the discussion in *The Lancet* Series on Midwifery which contextualised the varied settings around the world in which midwifery care is provided. For example, Walker et al.’s Mexican study [29] employed obstetric nurses as well as midwives to provide rural practice care. Wu et al.’s study in China [30] was carried out in areas where there had previously been no organised systematic antenatal care, and against a backdrop of political and socio-economic change. It was the only study in this category not to claim some benefit from the intervention. Both Walker et al. and Wu et al. focussed more on task-based work than on continuity of care. By contrast the other studies in this category occurred in the UK and Australia where there is a strong tradition of both systematic antenatal care and of midwifery practice, and all involved continuity of care: team midwifery [28] and caseload midwifery [31, 32]. Other midwifery-led interventions did have eligibility criteria based on specific risk factors, and are considered under the Restricted ‘lower risk’-based model section below.

#### Reduced or flexible visit interventions

There were four studies within this category; we note that several other similar studies did apply eligibility criteria based on risk, and these are considered under ‘Restricted lower-risk-based provision’. Two Zimbabwean studies addressed efficient use of limited resources. Majoko et al. [33] compared five visits to the standard 13. Clement et al. [34] – the oldest study - reported on an intervention where the reduction was from 13 to seven visits. Villar et al.’s [35] multinational study concluded that a reduced visit schedule could be implemented without adversely affecting clinical outcomes, although maternal satisfaction was reduced. We note, incidentally, that the new WHO guidelines on antenatal care, based on Vogel et al.’s secondary analysis [36], have increased the minimum number of recommended visits from four to eight [13].

#### Group-based care

There were two recent studies in this category, both comparing ‘group antenatal care’ with individual care. Jafari et al. [37] reported positive clinical outcomes such as the reduced likelihood of caesarean delivery and time to diagnosis for hypertension and urinary/vaginal infections. Satisfaction levels were significantly improved in the intervention group. Comparable clinical outcomes were not reported by Andersson et al. [38]; in their paper satisfaction with group care was reported in terms of there being “fewer deficiencies”.

Homer et al.’s associated SR [39] included two additional group care trials ([40, 41]) which we classified by their target group (adolescents and ‘women in the military/military wives’) rather than by the intervention type (see Table 6).

#### Multiple foci

We identified one systematic review which examined a range of interventions intended to reduce one specific outcome (stillbirth) [42]. These interventions included reduced visit schedules.

### Restricted ‘lower risk’-based model

We have distinguished certain types of intervention which were shared between our Universal and Restricted ‘lower risk’-based provision models (Table 4). However, while the format of the intervention was similar the target population was different. The Restricted ‘lower risk’-based studies comprised midwifery-led trials and reduced or flexible visit interventions. See reference lists T4-1 to T4-3 (Additional file 1).

#### Midwifery-led interventions

Twenty papers reported 12 ‘midwifery-led’ studies, mostly in Australia (5) and the UK (3), which placed risk eligibility criteria on potential participants (Table 3). In terms of timeframe they range from Flint’s 1989 study [43] to the new millennium. We have distinguished these from the midwifery-led interventions in the ‘Universal provision’ model because while the care given was often comparable, there was a crucial restriction on eligibility. Risk assessment in clinical practice now covers not just clinical (physical and/or psychological) factors, but social factors too, making it increasingly difficult for a pregnant woman to avoid being labelled as ‘not low risk’.

The Chinese study [44] took place in an environment without a strong tradition of midwifery care, differing, for example, from Australia and the UK. There was one economic analysis of a ‘midwife-led’ hospital antenatal clinic ([45]); the others all evaluated continuity intervention. Turnbull et al. ([46]) and McLachlan et al. ([47]) adopted a caseload approach; the remainder used a team midwifery approach.

There were five associated SRs, three of which also covered the ‘Universal provision’ midwifery-led interventions discussed above. As with the ‘midwifery-led’ category these covered studies in both our Universal and Restricted ‘lower risk’-based provision models.

#### Reduced or flexible visit interventions

As above in the Universal provision section, these six trials (four UK-based; two US-based) also investigated women’s satisfaction in addition to clinical outcomes. Chronologically, they were quite close together (published 1996–2000). Women’s satisfaction levels were lower in three studies ([48–50]). Only one study showed a slight increase in satisfaction ([51]), although the sample size was very small (*n* = 43 intervention, *n* = 38 control). In Sikorski et al.’s study [48], dissatisfaction was assumed if the women would have preferred more visits in the intervention arm, or fewer visits in the control arm. It should be noted that reduced visit schemes have different connotations worldwide. The previous reduced visit model suggested by the WHO of at least four visits [52], for example, was usually only applied in low-income countries. The studies reported here were all in high-income countries, where the model tends to have a baseline for all women with increased visits for those with complications. The early trials in the 1990s started from a base of a higher number of antenatal visits (often up to 14); what was termed ‘reduced’ visits (ranging from six for multiparous women [48] to eleven [53]) is now close to the norm in high-income countries.

Three of the four SRs ([54–56]) agreed that reduced visits did not result in detrimental clinical outcomes. Dowswell et al. [57] cautioned that if implemented in settings with limited resources, and where the number of visits was already low, further reductions might have a negative impact on perinatal mortality.

#### Multiple foci

As with the Universal Provision model, one SR examined a range of interventions aimed at reducing stillbirth, including one community-based midwifery-led intervention [42].

### Augmented provision model

Trials within this model comprised additional care interventions and behavioural interventions that supplemented routine care (Table 5). See reference lists T5-1 and T5-2 (Additional file 1).

Interventions in Table 5 were ‘universal’ as they did not restrict eligibility based on risk, but standard antenatal care was augmented by additional/specific clinical, educational or behavioural interventions. We distinguish these from behavioural interventions that were targeted at ‘higher risk’ groups (such as obese women, or smokers), which fit our Targeted ‘higher risk’-based model.

#### Additional care interventions

These 13 trials mainly involved the intervention of an educational element of care, and focussed on diverse outcomes, such as epidural use ([58]), low birth weight ([59]), perceived breast feeding advice and support ([60]), infant feeding practices ([61]), general attitudes and behaviour ([62]) and health service use in the year after birth ([63]). There were therefore no intervention and outcome commonalities for comparison. One SR concluded that telephone support may assist in reducing low birth weight, postnatal depressive symptoms and smoking relapse, as well as increasing breastfeeding duration ([64]). However, the authors caution that there was diversity in the nature of the interventions in the small number of studies available for comparison. In chronological terms Elbourne’s 1987 study ([62]) was an outlier: all others were published between 2005 and 2013.

#### Behavioural interventions

These seven studies covered diverse interventions, including measures to address issues such as gestational weight gain ([65]), anaemia [66], and self-hypnosis [67] to improve the childbirth experience. The oldest date from 2009 [65, 66]. The interventions were integrated into the core care package, and available to all women, not only those with a specific need. For example, the two studies by Barakat et al. [66, 68] recruited healthy pregnant women to an exercise programme. The exercise programme trials by Stafne et al. [69] and Barakat et al. [68] also reported on gestational diabetes mellitus, but were not able to influence this outcome positively.

The Ruifrok et al. SR [70] included the earlier of the Barakat studies and Phelan et al. [71], as well as studies we list under Targeted ‘higher risk’-based provision. The Domenjoz et al. SR [72] covered 16 studies, only three of which we had identified. This discrepancy occurred because many of their included studies were separate from and not additional to antenatal care, and therefore did not report any pregnancy outcomes. They were thus not eligible for our review. One associated systematic review [73] (since updated) did not cover any of our identified papers, an anomaly explained by the eclectic nature of behavioural interventions and the inclusion criteria for meta-analyses. Sibley et al. [74] included Azad et al. [75] which we listed under ‘Community delivered interventions’ (see Table 3).

### Targeted ‘higher risk’-based model

The review identified 67 trials that detailed risk-assessed targeted interventions. By ‘targeted’ here we mean that particular populations were targeted on the basis of some ‘higher risk’ calculation, not that particular outcomes were sought. This is a complex group of interventions, involving many different categories; what they have in common is that a particular risk-based factor was the principal criterion for trial eligibility. These trials are detailed in Table 6. See reference lists T6-1 to T6-7 (Additional file 1).

Population sub-groups with defined clinical, psychosocial and/or socio-demographic risk factors were the focus of these interventions, which ranged from managing anxiety and mental health issues, to reducing tobacco and alcohol consumption. Such a broad category defies easy classification, but it was noticeable that some sub-groups had temporal associations. For example, all but one of the ten studies aimed at trying to prevent preterm birth were published from 1990 to 2000, whereas the five studies focussing on obesity were all published after 2010.

We appreciate that pregnant women may have multiple risk factors and/or be at risk of more than one poor outcome; where possible we used the primary outcome identified by the authors, but in nine cases we had to label this group as ‘multiple risks’; there were three SRs associated with ‘multiple risks’. Other named risk factors ranged from risk of preterm labour or having a low birthweight baby to smoking, mental health issues, being overweight or being at risk of abuse.

Socio-demographic risk groups included adolescents, women on low income and women in or with partners in the armed forces.

## Discussion

In this scoping review we have identified many different trials of antenatal care which have been attempted around the world. Synthesising the evidence from such diverse studies is inevitably difficult: the context around the world varies widely, and has itself shifted over time within particular locations. Our taxonomy of Universal, Restricted ‘lower risk’-based, Augmented and Targeted ‘higher risk’-based provision models summarises the nature and intention of the various trials of antenatal care that have been conducted since the 1980s. It is therefore of assistance in answering questions about who is included in trials, the nature of the intervention, and what outcomes the trial hopes to achieve. It is possible that this taxonomy misses certain crucial characteristics that could differentiate models of antenatal care. However, our taxonomy allows us to present the data in a systematic way, and to highlight the range of foci behind these interventions, something we believe will be of interest to service designers and those planning further antenatal care model research. As noted in the introduction, this project has been the first step of a planned programme of research exploring the effects of different care models on pregnancy outcomes. We acknowledge that by focussing only on RCTs and SRs of RCTs, we have not included studies using other methodologies. Scoping studies may choose to select a broad range of research and non-research evidence - they are not limited to a single approach [76, 77]. Our decision to include only RCTs and SRs of RCTs means that our taxonomy is of models with existing evidence of effectiveness. We also wish to emphasise that while we would have preferred to use an alternative concept to ‘risk’ in our taxonomy this was inescapable given the approach adopted by many of the studies we analysed. We are aware of the dangers of reifying a risk-based approach with such terminology, and suggest that wherever possible future trials also test Universal models.

We acknowledge that the study and analysis of Universal models is large, as they are eclectic. Many of the recent examples were community-based models in low resource settings, reflecting the desire of health care providers in such countries to provide broad coverage for pregnant women. It might be argued that countries with more developed maternity care systems are able to provide a range of models, and that the Universal model is particularly suited to countries at a particular stage in their development. However, the fact that some countries with a long-standing history of midwifery care (UK, Australia, Sweden) also trialled Universal midwifery-led models suggests that the broad scope of Universal models could work globally. The same three high income countries – as well as several others – predominated in the Restricted ‘lower risk’-based model. Despite being well evaluated, extending or replicating such models is problematic, not least because the inherent causal mechanisms have not been identified. Nevertheless, exploring such models may offer the opportunity to identify these mechanisms. This is work in which the McTempo collaboration is currently engaged.

Any maternity care system must be flexible, and the proliferation of lifestyle-related morbidity such as obesity or diabetes means that augmented and targeted models will also be required. We have distinguished these models on the basis of their intended audience, but as we conceded earlier, there are inevitably areas of overlap within our taxonomy.

### Descriptions of the trials

The process of categorisation of these studies revealed an important limitation. While many of the reports described the intervention involved (i.e. how it was organised), they rarely detailed exactly what was done. It may be that their authors took it for granted that readers would understand what is involved in clinical care, but many did not discuss details of assessment and screening, or mention how the workforce was organised, or their philosophy of care – i.e. woman-centred, avoiding unnecessary interventions, optimising physiological processes, etc. Hoffman et al. [23] note that “the quality of description of interventions in publications… is remarkably poor” (page 1). In particular, the interaction between practitioner and pregnant woman - the human element - was rarely described. There is research indicating that women value how clinical procedures are carried out [78], and this is an area for future research. Authors may also have considered that readers would know what ‘routine care’ meant (i.e. the care given to the control arm in the trial), but as ‘routine care’ varies around the world and has changed over time this lack of information makes it difficult to know exactly what was different about the intervention. Also missing in many reports is a consideration of the underpinning values and philosophy of the intervention (noted by Hoffman et al. [23]), again perhaps because the authors assumed this was understood. This gap in the evidence makes replicating interventions more difficult, a significant hindrance when trying to evaluate and implement best evidence.

### Distinguishing and comparing the models

Establishing a taxonomy from 130 very different trials which all had multiple features entailed detailed discussion within the McTempo group. Our decision to use the trials’ intended population rather than their type of intervention as the principal (but not sole) means of categorising them was the most efficient way of teasing out divergent trials which might otherwise be categorised together.

There was considerable overlap between our categories in terms of the kind of intervention that was introduced: ‘midwifery-led’ and ‘reduced/flexible visit’ studies were at times universally available, and at times restricted. As their target population was different we felt it important to distinguish them since risk assessment clearly plays a significant role within health care. This is considered further below. This was most obviously seen in the Targeted ‘higher risk’-based provision model, where the interventions focused on those with identified risk criteria.

Some interventions could be of benefit in more than one model. For example, we placed trials designed to reduce the risk of gestational diabetes in the Targeted ‘higher risk’-based model as there was a specific pregnancy-focussed aim to these studies. However, there were similarities with some of the ‘Behavioural Intervention’ studies which focused on lifestyle issues such as weight reduction. Our review found that the combined effects of lifestyle programmes, when incorporated as part of routine antenatal care, may help to reduce gestational diabetes, gestational weight gain, and unplanned caesarean sections. There is scope for investigating such interventions further.

Group interventions may offer more peer support opportunities, as well as improving clinical outcomes, and are therefore worth investigating further as a means of delivering antenatal care, perhaps in conjunction with reduced individual visits. The ‘social’ element within such interventions sets them apart from traditional ‘one-to-one’ care as experienced in many high-income countries; if the claimed improvements in outcomes are repeated in further robust evaluations then this element is worthy of serious consideration. An example of this approach is the CenteringPregnancy™ Group Prenatal Care Model, evaluations of which suggest that outcomes are significantly improved even in populations which usually have poorer than average outcomes, such as teenage mothers and those from deprived backgrounds [79, 80]. The multicentre RCT by Ickovics et al. [81] was published too late for inclusion in our search, and we are aware of another recently commenced RCT evaluating CenteringPregnancy™ in the USA. Other group antenatal care schemes have also been evaluated [19, 82].

The heterogeneity of the studies included in this review is also reflected in the broad range of primary outcome measures. Even within the studies examining midwifery-led care, the primary outcomes ranged from the process of care (continuity of caregiver, number of intrapartum caregivers, known caregiver at birth, overall clinic salary costs) to health-related outcomes (preterm birth, various indices of morbidity, psychological well-being) and patient preference-related outcomes (satisfaction with care). Certain outcomes (e.g. satisfaction) were often measured using study-specific questionnaires, making comparisons problematic. Indeed, the heterogeneity of the studies we identified means that systematic review and meta-analysis is often not possible across a range of outcomes. Nevertheless, we did identify scope to conduct a meta-analysis of neonatal outcomes.

### Risk considerations

While interventions in the Universal provision model offered care that was meant to be available to everyone, interventions in the Restricted ‘lower risk’-based model were limited to women deemed low risk. However, the definition of ‘low risk’ varied between studies, which makes comparisons much harder. Indeed, the whole notion of risk labelling is problematic [83], and the practice may at times even be counter-productive [84], not least because risk assessment has evolved and grown. As noted above it includes social factors too, so it is harder for a woman to avoid the label ‘not low risk’. McCourt et al.’s [85] exploration of women’s views about research priorities found that this focus on risk identification was causing concern. As long ago as 1989, in the first edition of *Effective Care in Pregnancy and Childbirth,* concerns were being raised that the over-use of risk labels could predispose pregnant women to poorer outcomes [86]. Renfrew et al. [11] suggest that, rather than concentrating on risk identification and management, the primary focus of both care and research should be on optimising biological, psychological, social and cultural processes, and tailoring care to the needs of individual women and infants, thereby avoiding viewing pregnancy and childbirth through a ‘risk lens’. They offer the QMNC framework as a means of examining the factors underpinning these processes, an approach that could be used in planning antenatal care interventions. The paper proposes a distinction between ‘what all women and babies need’ and ‘what some women and babies also need’, and suggests using the terms ‘healthy women and babies’ and ‘women and babies with complications’ rather than referring to high and low risk criteria. While the use of the term ‘risk’ proved inescapable within this taxonomy of studies to date, we would advocate that future interventions and models resist the high-versus-low risk dichotomy, and instead use the terminology proposed by Renfrew et al. – e.g. “skilled supportive and preventive care for all, promotion of normal reproductive processes, first-line management of complications, and skilled emergency care” [11].

### Geographical and historical considerations

Including only English-language articles may have missed some interventions from non-Anglophone countries. However, in addition to countries in which English is the predominant language or at least widely used (Australia, Canada, India, Ireland, Kenya, Malawi, Pakistan, South Africa, UK, USA, Zambia, Zimbabwe) we identified studies from Asia (China, Bangladesh, Indonesia, Nepal, Thailand, Vietnam), Francophone Africa (Benin), the near East (Iran, Saudi Arabia), Latin America and the Caribbean (Argentina, Cuba, Chile, Guatemala, Mexico), and across Europe (Denmark, Finland, France, Germany, the Netherlands, Norway, Spain, Sweden). The countries listed here also indicate that while the research infrastructure required to conduct and report robust RCTs in widely-available journals may be a source of bias for researchers in some developing countries, this is by no means an inevitable obstacle. The community-delivered interventions (Table 3) were almost exclusively in low-income countries. Our review agrees with Persson et al. [87] and Prost et al. [88] that community interventions show promise for outcome benefits in areas with limited resources (e.g. rural Asia). Lassi et al.’s [89] recent Cochrane review found that such schemes reduce neonatal mortality and morbidity as well as maternal morbidity.

It was noticeable that of the 67 trials in the Targeted ‘higher risk’-based model, 37 were based in the USA. Midwifery does not have a long tradition there (indeed, in some parts it is still unknown). Much of the focus of US research has been on reducing the likelihood of poor outcomes (notably low birth weight or preterm birth) within specific populations; those on low incomes, African Americans and Native Americans featured prominently.

The taxonomy also allows us to review historical trends. The Restricted ‘lower risk’-based model spans the longest period, from the 1980s to very recently, and these studies were predominantly from Australia and the UK. This reflects the particular place of midwifery within these countries, as well as historical concerns in the 1980s and 1990s about over-medicalisation of care and the role of choice and continuity of care within the maternity services. Offering ‘continuity models’, including team and caseload midwifery, was an attempt to improve both clinical outcomes and maternal satisfaction. However, what is striking about the interventions we have reviewed here is both how trends change over time, and how different countries apply different interventions. While the ‘midwifery-led’ interventions were a feature of the 1980s and 1990s in Australia and the UK, in more recent years they have featured in countries which have not had the same strong midwifery tradition, such as China and Mexico. The interventions tested there bore little resemblance to midwifery as experienced, for example, in the UK.

On the basis of this review, we propose that policy makers who are intending to amend current antenatal care, or to introduce it for the first time, should consider what type of care is most appropriate for their population, based on the findings from this study. We also propose that there is a need for a programme of research that examines different models of care from the perspective of what is known to constitute good quality care. This includes making explicit the philosophical underpinnings of proposed and existing models, and should also comprise stakeholder involvement in the development of a core outcomes data set so that comparative and observational studies in this area can be more easily compared.

## Conclusion

This comprehensive taxonomy provides an explanation of 130 antenatal care intervention trials from around the world over a period of 30 years. The trials were diverse in terms of purpose and scope, but we believe that clarifying this picture will help to inform thinking on future interventions. We can conclude that different antenatal care trials have claimed a range of improved clinical, psychosocial and organisational outcomes, but as yet the causal mechanisms are not understood, and this is an area that must be addressed if best evidence is to be replicated and extended. Our taxonomy of ‘Universal’ provision, ‘Restricted ‘lower-risk’-based’ provision, ‘Augmented’ provision and ‘Targeted ‘higher-risk’-based’ provision models may also help decision makers, service planners and policy makers in planning new strategies, although, as we have noted, we suggest that the focus is on inclusiveness rather than being focussed on inconsistent notions of risk. The focus on the intended target for an intervention is, we believe, the clearest way of explaining how and why trials in this field have been conducted. However, the field is dynamic, and since our search further trials have been initiated. We recommend that both policy makers and researchers in this area should use the findings of this review as a basis for decision making in this area in future.

## Additional file

Additional file 1: Articles and Systematic Reviews from Tables 3-6. (DOCX 40 kb)

## Abbreviations

HIV: Human Immunodeficiency Virus
IM: Independent midwife
McTempo: Models of care: the effects on maternal & perinatal outcomes
NHS: National Health Service
PRISMA: Preferred Reporting Items for Systematic Reviews and Meta-analyses
QMNC: Quality maternal and newborn care
RCT: Randomised controlled trials
SPIO: Study design, population, intervention, outcomes
SR: Systematic review
WHO: World Health Organisation
