# Supplementary material for: Antenatal care trial interventions: a systematic scoping review and taxonomy development of care models
Source: BMC Pregnancy Childbirth. 2017 Jan 6;17:8. doi: 10.1186/s12884-016-1186-3 (PMC5216531; doi:10.1186/s12884-016-1186-3)
Supplement: Additional file 1: — Articles and Systematic Reviews from Tables 3-6. (DOCX 40 kb) [file 12884_2016_1186_MOESM1_ESM.docx]

**Articles and Systematic Reviews from Tables 3-6**

**T3-1 Community delivered interventions – Universal provision model**

Azad K, Barnett S, Banerjee B 2010 Effect of scaling up women’s groups on birth outcomes in three rural districts in Bangladesh: a cluster-randomised controlled trial. Lancet 375: 1193-1202

Bhutta ZA, Soofi S, Cousens S, Mohammad S, Memon ZA, Ali I, Feroze A, Raza F, Khan A, Wall S, Martines J 2011 Improvement of perinatal and newborn care in rural Pakistan through community-based strategies: a cluster-randomised effectiveness trial. Lancet 377: 403-412

Colborn T, Nambiar B, Bondo A, Makwenda C, Tsetekani E, Makonda-Ridley A, Msukwa M, Barker P, Kotagal U, Williams C, Davies R, Webb D, Flatman D, Lewycka S, Rosato M, Kachale F, Mwansambo C, Costello A 2013 Effects of quality improvement in health facilities and community mobilization through women’s groups on maternal, neonatal and perinatal mortality in three districts of Malawi: MaiKhanda, a cluster randomized controlled effectiveness trial. International Health 5: 180-195

Darmstadt GL, Choi Y, Arifeen SE, Bari S, Rahman SM, Mannan I, Seraji HR, Winch PJ, Saha SK, Ahmed AS, Ahmed S, Begum N, Lee AC, Black RE, Santosham M, Crook D, Baqui AH 2010 Evaluation of a cluster-randomized controlled trial of a package of community-based maternal and newborn interventions in Mirzapur, Bangladesh. PLoS ONE 5: e9696

Fottrell E, Azad K, Kuddus A, Younes L, Shaha S, Nahar T, Aumon BH, Hossen M, Beard J, Hossain T, Pulkki-Brannstrom A-M, Skordis-Worrall J, Prost A, Costello A, Houweling TAJ 2013 The effect of increased coverage of participatory women’s groups on neonatal mortality in Bangladesh. JAMA Pediatrics 167 (9) 816-820

Jokhio AH, Winter HR, Cheng KK 2005 An intervention involving traditional birth attendants and perinatal and maternal mortality in Pakistan. New England Journal of Medicine 552 (20) 2091-2099

Lewycka S, Mwansambo C, Rosato M, Kazembe P, Phiri T, Mganga A, Chapota H, Malamba F, Kainja E, Newell ML, Greco G, Pulkki-Brannstrom AM, Skordis-Worrall J, Vergnano S, Osrin D, Costello A 2013 Effect of women’s groups and volunteer peer counselling on rates of mortality, morbidity, and health behaviours in mothers and children in rural Malawi (MaiMwana): a factorial, cluster-randomised controlled trial. Lancet 381: 1721-1735

Manandhar DS, Osrin D, Shrestha BP, Mesko N, Morrison J, Tumbahangphe KM, Tamang S, Thapa S, Shrestha D, Thapa B, Shrestha JR, Wade A, Borghi J, Standing H, Manandhar M, Costello AM 2004 Effect of a participatory intervention with women’s groups on birth outcomes in Nepal: cluster-randomised controlled trial. Lancet 364: 970-979

Midhet F, Becker S 2010 Impact of community-based interventions on maternal and neonatal health indicators: results from a community randomized trial in rural Balochistan, Pakistan. Reproductive Health 7 (30) 1-10

Miller PC, Rashida G, Tasneem Z, Haque MU 2012 The effect of traditional birth attendant training on maternal and neonatal care. International Journal of Gynaecology and Obstetrics 117:148-152

More NS, Bapat U, Das S, Alcock G, Patil S, Porel M, Vaidya L, Fernandez A, Joshi W, Osrin D 2012 Community mobilization in Mumbai slums to improve perinatal care and outcomes: a cluster randomized controlled trial. PLoS Medicine 9: e1001257

Mullany BC, Becker S, Hindin MJ 2007 The impact of including husbands in antenatal health education services on maternal health practices in urban Nepal: results from a randomised controlled trial. Health Education Research 22: 166-176

Pasha O, McClure EM, Wright LL, Saleem S, Goudar SS, Chomba E, Patel A, Esamai F, Garces A, Althabe F, Kodkany B, Mabeya H, Manasyan A, Carlo WA, Derman RJ, Hibberd PL, Liechty EK, Krebs N, Hambridge KM, Buekens P, Moore J, Jobe AH, Koso-Thomas M, Wallace DD, Stalls S, Goldenberg R,L, 2013 A combined community and facility-based aqpproach to improve pregnancy outcomes in low resource settings: a Global Network cluster randomized trial. BMC Medicine 11 (215) 1-12

Persson LA, Nga NT, Malqvist M, Thi Phuong Hoa D, Eriksson L, Wallin L, Selling K, Huy TQ, Duc DM, Tiep TV, Thi Thu Thuy V, Ewald U 2013 Effect of facilitation of local maternal-and-newborn stakeholder groups on neonatal mortality: cluster-randomized controlled trial. PLoS Medicine 10 e1001445

Prost A, Colbourn T, Seward N, Azad K, Coomarasamy A, Copas A, Houweling TAJ, Fottrell E, Kuddus A, Lewycka S, MacArthur C, Manandhar D, Morrison J, Mwansambo C, Nair N, Nambiar B, Osrin D, Pagel C, Phiri T, Pulkki-Brannstrom AM, Rosato M, Skordis-Worrall J, Saville N, More NS, Shrestha B, Tripathy P, Wilson A, Costello A 2013 Women’s groups practising participatory learning and action to improve maternal and newborn health in low-resource settings: a systematic review and meta-analysis. Lancet 381: 1736-1746

**T3-2 Midwifery led interventions – Universal provision model**

Devane D, Brennan M, Begley C 2010 Socioeconomic value of the midwife: a systematic review, meta-analysis, meta-synthesis and economic analysis of midwife-led models of care. Royal College of Midwives Trust, London

McLachlan BK, Rouse PM, Baines MA, Jones PW, Wyatt JC, Johanson RB (The North Staffordshire *Changing Childbirth* Research Team) 2000 A randomised study of midwifery caseload care and traditional ‘shared care’. Midwifery 16: 295-302

Rowley MJ, Hensley MJ, Brinsmead MW, Wlodarczyk JH 1995 Continuity of care by a midwife team versus routine care during pregnancy and birth: a randomised trial. Centre for Reviews and Dissemination, University of York

Sandall J, Soltani H, Gates S, Shennan A, Devane D 2013 Midwife-led continuity models versus other models of care for childbearing women. Cochrane Database of Systematic Reviews

Tracy SK, Hartz DL, Tracy MB, Allen J, Forti A, Hall B, White J, Lainchbury A, Stapleton H, Beckmann M, Bisits A, Homer C, Foureur M, Welsh A, Kildea S 2013 Caseload midwifery care versus standard maternity care for women of any risk: M@NGO, a randomised controlled trial. Lancet 382: 1723-1732

Waldenstrom U, Turnbull D 1998 *A systematic review comparing continuity of midwifery care with standard maternity services*. British Journal of Obstetrics and Gynaecology 105: 1160-1170

Walker D, DeMaria L, Gonzalez-Hernandez D, Padron-Salas A, Romero-Alvarez M, Suarez L 2013 Are all skilled birth attendants created equal? A cluster randomised controlled study of non-physician based obstetric care in primary health care clinics in Mexico. Midwifery 29:1199-1205

Wu Z, Viisainen K, Wang Y, Hemminki 2011 Evaluation of a community-based randomized controlled prenatal care trial in rural China. BMC Health Service Research 11 (92) 1-10

**T3-3 Reduced/flexible visit interventions - Universal provision model**

Carroli G, Villar J, Piaggio G, Khan-Neelofur D, Gulmezoglu M, Mugford M, Lumbiganon P, Farnot U, Bersgjo P 2001 WHO systematic review of randomised controlled trials of routine antenatal care. Lancet 357: 1565-1570

Clement S, Sikorski J, Wilson J, Das S, Smeeton N 1996 Women’s satisfaction with traditional and reduced antenatal visit schedules. Midwifery 12: 120-128

Majoko F, Munjanja SP, Nystrom L, Mason E, Lindmark G 2007 Randomised controlled trial of two antenatal care models in rural Zimbabwe. British Journal of Obstetrics and Gynaecology 114: 802-811

Munjanja SP, Lindmark G, Nystrom L 1996 Randomised controlled trial of a reduced-visits programme of antenatal care in Harare, Zimbabwe. Lancet 348: 364-369

Villar J, BA’aqeel H, Piaggio G 2001 WHO antenatal care randomised trial for the evaluation of a new model of routine antenatal care. Lancet 357: 1551-1564

**T3-4 Group-based antenatal care interventions – Universal provision model**

Andersson E, Christensson K, Hildingsson I 2013 Mothers’ satisfaction with group antenatal care vesus individual antenatal care – a clinical trial. Sexual and Reproductive Healthcare 4: 113-120

Homer CS, Ryan C, Leap N, Foureur M, Teate A, Catling_Paull C 2012 Group versus conventional antenatal care for women. Cochrane Database of Systematic Reviews

Jafari F, Eftekhar H, Fotouhi A, Mohammad K, Hantoushzadeh S 2010 Comparison of maternal and neonatal outcomes of group versus individual prenatal care: a new experience in Iran. Healthcare for Women International 31: 571-584 (See also: Jafari F, Eftekhar H, Mohammad K, Fotouhi A 2010 Does group prenatal care affect satisfaction and prenatal care utilization in Iranian pregnant women? Iranian Journal of Public Health 39 (2) 52-62)

**T3-5 Systematic review with multiple foci – Universal provision model**

Yakoob MY, Menezes EV, Soomro T, Haws RA, Darmstadt GL, Bhutta ZA 2009 Reducing stillbirths: behavioural and nutritional interventions before and during pregnancy. *BMC Pregnancy and Childbirth* 2009, **9**(Suppl 1):S3 doi:10.1186/1471-2393-9-S1-S3

New T4 lists, created from old T3

**T4-1 Midwifery led interventions - Restricted ‘lower risk’-based model**

Begley C, Devane D, Clarke M, McCann C, Hughes P, Reilly M, Maguire R, Higgins S, Finan A, Gormally S and Doyle M 2011 Comparison of midwife-led and consultant-led care of healthy women at low risk of childbirth complications in the Republic of Ireland: a randomised trial. BMC Pregnancy and Childbirth 11:85

Biro MA, Waldenstrom U, Panniflex JH 2000 Team midwifery care in a tertiary level obstetric service: a randomised control trial. Birth 26:3 168-173 (See also related paper from the same study: Biro MA, Waldenstrom U, Brown S, Panniflex JH 2003 Satisfaction with team midwifery care for low and high risk women: a randomized controlled trial. BIRTH 30 (1) 1-10)

Devane D, Brennan M, Begley C 2010 Socioeconomic value of the midwife: a systematic review, meta-analysis, meta-synthesis and economic analysis of midwife-led models of care. Royal College of Midwives Trust, London

Flint C, Poulengeris P, Grant A 1989 The know your midwife scheme: a randomised trial of continuity of care by a team of midwives. Midwifery 5:11-16

Giles W, Collins J, Ong F, MacDonald R 1992 Antenatal care of low risk obstetric patients by midwives: a randomised controlled trial. The Medical Journal of Australia. 157 (3) 158-161

Gu C, Wu X, Ding Y, Zhu X, Zhang Z 2013 The effectiveness of a Chinese midwives’ antenatal clinic service on childbirth outcomes for primiparae: a randomised controlled trial. International Journal of Nursing Studies 50 (2013) 1689-1697

Harvey S, Rach D, Stainton MC, Jarrell J, Brant R 2002 Evaluation of satisfaction with midwifery care. Midwifery 18: 260-267

Hicks C, Spurgeon P, Barwell F 2003 Changing childbirth: a pilot project. Journal of Advanced Nursing 42 (6) 617-628

Homer CSE, Davis GK, Brodie PM, Sheehan A, Barclay LM, Wills J, Chapman MG 2001 Collaboration in maternity care: a randomised controlled trial comparing community-based continuity of care with standard hospital care. British Journal of Obstetrics and Gynaecology 108: 16-22 (See also: Homer CS, Matha DV, Jordan LG, Wills J, Davis GK 2001Community-based continuity of midwifery care versus standard hospital care: a cost analysis. Australian Health Review 24 (1) 85-93)

Khan-Neelofur D, Gulmezoglu M, Villar J 1998 Who should provide routine antenatal care for low-risk women, and how often? A systematic review of randomised controlled trials. WHO Antenatal Care Trial Research Group. Paediatric and Perinatal Epidemiology. 12 (2) 118-122

McLachlan HL, Forster DA, Davey MA, Farrell T, Gold L, Biro MA, Flood M, Oats J, Waldenstrom U 2012 Effects of continuity of care by a primary midwife (caseload midwifery) on caesarean section rates in women of low obstetric risk: the COSMOS randomised controlled trial. British Journal of Obstetrics and Gynaecology 25 July: 1483-1492

Sandall J, Soltani H, Gates S, Shennan A, Devane D 2013 Midwife-led continuity models versus other models of care for childbearing women. Cochrane Database of Systematic Reviews

Turnbull D, Holmes A, Shields N, Cheyne H, Twaddle S, Gilmour WH, McGinley M, Reid M, Johnstone I, Geer I, McIIwaine G, Lunan CB 1996 Randomised, controlled trial of efficiency of midwife-managed care. Lancet 348:213-218 (See also: Turnbull D, Shields N, McGinley M, Holmes A, Cheyne H, Reid M, Young D, Gilmour WH 1999 Can midwife-managed units improve continuity of care? British Journal of Midwifery 7 (8) 499-503 – and - Shields N, Turnbull D, Reid M, Holmes A, McGinley M, Smith LN 1998 Satisfaction with midwife-managed care in different time periods: a randomised controlled trial of 1299 women. Midwifery 14: 85-93 – and - Young D, Shields A, Holmes A, Turnbull D, Twaddle S 1997 A new style of midwife-managed antenatal care: costs and satisfaction. British Journal of Midwifery 5 (9) 540-545)

Villar J, Carroli G, Khan-Neelofur D, Piaggio G, Gulmezoglu M 2007 Patterns of routine antenatal care for low-risk pregnancy. The Cochrane Database of Systematic Reviews

Waldenstrom U, Nilsson C-A 1994 Experience of childbirth in birth center care. Acta Obstetricia et Gynecologica Scandinavica 73: 547-554 (See also: Waldenstrom U, Nilsson C-A 1997 A randomized controlled study of birth center care versus standard maternity care: effects on women’s health. BIRTH 24 (1) 17-26 – and - Waldenstrom U, Nilsson C-A, Winbladh B 1997 The Stockholm birth centre trial: maternal and infant outcome. British Journal of Obstetrics and Gynaecology 104: 410-418)

Waldenstrom U, Turnbull D 1998 *A systematic review comparing continuity of midwifery care with standard maternity services*. British Journal of Obstetrics and Gynaecology 105: 1160-1170

Waldenstrom U, Brown S, McLachlan H, Forster D, Brennecke S 2000 Does team midwife are increase satisfaction with antenatal, intrapartum, and postpartum care? A randomized controlled trial. BIRTH 27 (3) 156-167 (See also: Waldenstrom U, McLachlan H, Forster D, Brennecke S, Brown S 2001 Team midwife care: maternal and infant outcomes. Australian and New Zealand Journal of Obstetrics and Gynaecology 41(3) 257-264)

**T4-2 Reduced/flexible visit interventions - Restricted ‘lower risk’-based model**

Carroli G, Villar J, Piaggio G, Khan-Neelofur D, Gulmezoglu M, Mugford M, Lumbiganon P, Farnot U, Bersgjo P 2001 WHO systematic review of randomised controlled trials of routine antenatal care. Lancet 357: 1565-1570

Dowswell T, Carroli G, Duley, Gates S, Gulmezoglu AM, Khan-Nellofur D, Piaggio G 2010 Alternative versus standard packages of antenatal care for low-risk pregnancy. Cochrane Database of Systematic Reviews

Henderson J, Roberts T, Sikorski J, Wilson J, Clement S 2000 An economic evaluation comparing two schedules of antenatal visits. Journal of Services Health Research and Policy 5: 69-75

Jewell D, Sharp D, Sanders J, Peters TJ 2000 A randomised controlled trial of flexibility in routine antenatal care. British Journal of Obstetrics and Gynaecology 107:1241-1247

Khan-Neelofur D, Gulmezoglu M, Villar J 1998 Who should provide routine antenatal care for low-risk women, and how often? A systematic review of randomised controlled trials. WHO Antenatal Care Trial Research Group. Paediatric and Perinatal Epidemiology. 12 (2) 118-122

McDuffie RS, Beck A, Bischoff K, Cross J, Orleans M 1996 Effect of frequency of prenatal care visits on perinatal outcome among low-risk women. A randomized controlled trial. JAMA 275: 847-851 (See also: McDuffie RS, Bischoff K, Cross J, Beck A 1995 An evaluation of risk-based prenatal care: a randomized controlled trial. AJOG 172: 270)

Sikorski J, Wilson J, Clement S, Das S, Smeeton N 1996 A randomised control ed trial comparing two schedules of antenatal visits: the antenatal care project. BMJ 312: 546-553

Tucker JS, Hall MH, Howie PW, Reid ME, Barbour RS, Florey CD, McIlwaine GM 1996 Should obstetricians see women with normal pregnancies? A multicentre randomised controlled trial of routine antenatal care by general practitioners and midwives compared with shared care led by obstetricians. BMJ 312: 554-559

Villar J, Carroli G, Khan-Neelofur D, Piaggio G, Gulmezoglu M 2007 Patterns of routine antenatal care for low-risk pregnancy. The Cochrane Database of Systematic Reviews

Walker DS, Koniak-Griffin D 1997 Evaluation of a reduced-frequency prenatal visit schedule for low-risk women at a free-standing birthing center. Journal of Nurse-Midwifery 42: 295-303

**T4-3 Systematic review with multiple foci - Restricted ‘lower risk’-based model**

Yakoob MY, Menezes EV, Soomro T, Haws RA, Darmstadt GL, Bhutta ZA 2009 Reducing stillbirths: behavioural and nutritional interventions before and during pregnancy. Cochrane Database of Systematic Reviews

**T5-1 Additional care interventions - Augmented provision model**

Au F, Shiell A, Van Der Pol M, Johnston DW, Tough S 2006 Does supplementary prenatal nursing and home visitation reduce healthcare costs in the year after childbirth? Journal of Advanced Nursing 56: 657-668

Bergstrom M, Keiler H, Waldenstrom U 2009 Effects of natural childbirth preparation versus standard antenatal education on epidural rates, experience of childbirth and parental stress in mothers and fathers: a randomised controlled multicentre trial. British Journal of Obstetrics and Gynaecology 116: 1167-1176

Brown HC, Smith HJ 2004 Giving women their own case notes to carry during pregnancy. Cochrane Database of Systematic Reviews

Dennis CL, Kingston D 2008 A systematic review of telephone support for women during pregnancy and the early postpartum period. Journal of Obstetric, Gynecology and Neonatal Nursing 37: 301-314

Ekstrom A, Widstrom A-M, Nissen E 2006 Does continuity of care by well-trained breastfeeding counsellors improve a mother’s perception of support? Birth 33: 123-130

Ekhtiari YS, Majlessi F, Foroushani AR, Shakibazadeh E 2014 Effect of a self-care educational program based on the health belief model on reducing low birth weight among pregnant Iranian women. International Journal of Preventative Medicine 5: 76-82

Elbourne D, Richardson M, Chalmers I 1987 The Newbury Maternity Care Study: a randomised controlled trial to assess a policy of women holding their own obstetric records. British Journal of Obstetrics and Gynaecology 94: 612-619

Hajian S, Shariati M, Najmabadi KM, Yunesian M, Ajami ME 2012 The effect of the Extended Parallel Process Model of childbirth education for decreasing the rate of Caesarean section among Iranian women. Life Science Journal 9: 445-452

Hemminki E, Long Q, Zhang W_H, Wu Z, Raven J, Tao F, Yan H, Wang Y, Klemetti R, Zhang T, Regushevskaya E, Tang S 2013 Impact of financial and educational interventions on maternity care: results of cluster randomized trials in rural China. Maternal and Child Health Journal 17:208-221

Jennings L, Yebadokpo AS, Affo J, Agbogbe M 2010 Antenatal counselling in maternal and newborn care: use of job aids to improve health worker performance and maternal understanding in Benin. BMC Pregnancy and Childbirth 10: 75

Leung Ss, Lam TH 2012 Group antenatal intervention to reduce perinatal stress and depressive symptoms related to intergenerational conflicts: a randomized controlled trial. International Journal of Nursing Studies 49: 1391-1402

Nsibande D, Doherty T, Ijumba P, Tomlinson M, Jackson D, Sanders D, Lawn J 2013 Assessment of the uptake of neonatal and young infant referrals by community health workers to public health facilities in an urban informal settlement., KwaZulu-Natal, South Africa. BMC Health Services Research 13: 47

Nuraini E, Parker E 2005 Improving knowledge of antenatal care (ANC) among pregnant women: a field trial in central Java, Indonesia. Asia-Pacific Journal of Public Health 17: 3-8

Svensson J, Barclay L, Cooke M 2009 Randomised-controlled trial of two antenatal education programmes. Midwifery 25: 114-125

Tough SC, Johnston DW, Siever JE, Jorgenson G, Slocombe L, Lane C, Clarke M 2006 Does supplementary prenatal nursing and home visitation support improve resource use in a universal health care system? A randomized controlled trial in Canada. Birth 33: 183-194

**T5-2 Behavioural interventions - Augmented provision model**

Asbee SM, Jenkins TR, Butler JR, White J, Elliot M, Rutledge A 2009 Preventing excessive weight gain during pregnancy through dietary and lifestyle counselling: a randomized controlled trial. Obstetrics and Gynecology 113: 305-312

Barakat R, Ruiz JR, Lucia A 2009 Exercise during pregnancy and risk of maternal anaemia: a randomised controlled trial. British Journal of Sports Medicine 43: 954-956

Barakat R, Pelaez M, Lopez C, Lucia A, Ruiz JR, 2013 Exercise during pregnancy and gestational diabetes-related adverse effects: a randomised controlled trial. British Journal of Sports Medicine 47: 630-636

Domenjoz I, Kayser B, Boulvain M 2014 Effect of physical activity during pregnancy on mode of delivery. American Journal of Obstetrics and Gynecology 401:e1-11

Ota E, Tobe-Gai R, Mori R, Farrar D 2012 Antenatal dietary advice and supplementation to increase energy and protein intake. Cochrane Database of Systematic Reviews

Phelan S, Phipps MG, Abrams B, Darroch F, Schaffner A, Wing RR 2011 Randomized trial of a behavioral intervention to prevent excessive gestational weight gain: the Fit for Delivery study. American Journal of clinical Nutrition 93: 772-779

Rakhshani A, maharana S, Raghuram N, Nagendra HR, & Venkatram P 2010 Effects of integrated yoga on quality of life and interpersonal relationship of pregnant women. *Quality of Life Research,* 19**,** 1447-1455

Ruifrok AE, Van Poppel MN Van Wely M, Rogozinska E, Khan KS, Groot CJM, Thangaratinam S, Mol BW 2014 Association between weight gain during pregnancy and pregnancy outcomes after dietary and lifestyle interventions: a meta-analysis. American Journal of Perinatology 31: 353-364

Sibley LM, Sipe TA, Brown CM, Diallo MM, McNatt K, Habarta N 2012 Traditional birth attendant training for improving health behaviours and pregnancy outcomes. Cochrane Database of Systematic Reviews

Stafne SN, Salvesen KA, Romundstad PR, Eggebo TB, Carlsen SM, Morkved S 2012 Regular exercise during pregnancy to prevent gestational diabetes. Obstetrics and Gynecology 119(1) 29-36

Werner A, Uldbjerg N, Zachariae R, Wu CS, Nohr EA 2013 Antenatal hypnosis training and childbirth experience: a randomized controlled trial. Birth 40 (4) 272-280

**T6-1 Interventions for women with various or multiple risks - Targeted ‘higher risk’-based model**

Blondel B, Breart G 1992 Home visits for pregnancy complications and management of antenatal care: an overview of three randomised controlled trials. British Journal of Obstetrics and Gynaecology 99: 283-286

Blondel B, Breart G 1995 Home visits during pregnancy: consequences on pregnancy outcome, use of health services, and women’s situations. Seminars in Perinatology 19: 263-271

Brooten D, Youngblut JM, Brown L, Finker SA, Neff DF, Madigan E 2001 A randomised trial of nurse specialist home care for women with high-risk pregnancies: outcome and cost. American Journal of Managed Care 7 (9) 793-803

Dawson A, Middlemiss C, Coles EC, Gough NA, Jones ME 1989 A randomised study of a domiciliary antenatal care scheme: the effect on hospital admissions. British Journal of Obstetrics and Gynaecology 96: 1319-1322 (See also: Middlemiss C, Dawson AJ, Gough N, Jones ME, Coles EC (1989) A randomised study of a domiciliary antenatal care scheme: maternal psychological effects. Midwifery, 5, 69-74)

Dawson A, Cohen D, Candelier C, Jones G, Sanders J, Thompson A, Arnall C, Coles E 1999 domiciliary midwife support in high-risk pregnancy incorporating telephonic fetal heart rate monitoring: a health technology randomized assessment. Journal of Telemedicine and Telecare 5:220-230

Dowswell T, Middleton P, Weeks A 2009 Antenatal day care units versus hospital admission for women with complicated pregnancy. Cochrane Database of Systematic Reviews

El Mohandes AAE, Kiely M, Gantz MG, El-Khorazaty MN 2011 Very preterm birth is reduced in women receiving an integrated behavioural intervention: a randomized controlled trial. Maternal and Child Health Journal 15: 19-28

Kemp L, Harris E, McMahon C, Matthey S, Vimpani G, Anderson T, Schmied V, Aslam H, Zapart S 2011 Child and family outcomes of a long-term nurse visitation programme: a randomised controlled trial. Archives of Disease in Childhood 96: 533-540 (See also: Kemp L, Harris E, McMahon C, Matthey S, Vimpani G, Anderson T, Schmied V, Aslam H 2013 Benefits of psychosocial intervention and continuity of care by child and family health nurses in the pre- and postnatal period: process evaluation. Journal of Advanced Nursing 69: 1850-1861)

Klerman LV, Ramey SL, Goldenberg RL, Marbury S, Hou J, Cliver SP 2001 A randomized trial of augmented prenatal care for multiple-risk, Medicaid-eligible African American women. American Journal of Public Health 91: 105-111

Lee E, Mitchell-Herzfeld SD, Lowenfels AA, Greene R, Dorabawila V, Dumont KA 2009 Reducing low birth weight through home visitation: a randomized controlled trial. American Journal of Preventative Medicine 36: 154-160

Turnbull DA, Wilkinson C, Gerard K, Shanahan M, Ryan P, Griffith EC, Kruzins G, Stamp GE 2004 Clinical, psychosocial, and economic effects of antenatal day care for three medical complications of pregnancy: a randomised controlled trial of 395 women. Lancet 363: 1104-1109 (See also: Turnbull DA, Wilkinson C, Griffith EC, Kruzins G, Gerard K, Shanahan M, Stamp GE (2006) The psychosocial outcomes of antenatal day care for three medical complications of pregnancy: a randomised controlled trial of 395 women. *Australian & New Zealand Journal of Obstetrics & Gynaecology,* 46**,** 510-6

Villar J, Farnot U, Barros F, Victora C, Langer A, Belizan JM 1992 A Randomized trial of psychosocial support during high-risk pregnancies. The New England Journal of Medicine 327: 1266-1271

**T6-2 Interventions for women at risk of preterm birth or having a low birthweight baby - Targeted ‘higher risk’-based model**

Bryce RL, Stanley FJ, Garner JB 1991 Randomized controlled trial of antenatal social support to prevent preterm birth. British Journal of Obstetrics and Gynaecology 98: 1001-1008

Depp R, Iams JD, Goldenberg R, Key T, Entman S, Laros RK, Creasy RK, Heilbron D 1993 Multicenter randomized, controlled trial of a preterm birth prevention program. American Journal of Obstetrics and Gynecology 169: 352-366

Heins HC, Nance NW, McCarthy BJ, Efird CM 1990 A randomized trial of nurse-midwifery prenatal care to reduce low birth weight. Obstetrics and Gynecology 75: 341-345

Hodnett ED, Fredricks S, Weston J 2010 Support during pregnancy for women at increased risk of low birthweight babies. Cochrane Database of Systematic Reviews

Langer A, Victora C, Victora M, Barros F, Farnot U, Belizan J, Villar J 1993 The Latin American trial of psychosocial support during pregnancy: a social intervention evaluated through an experimental design. Social Science and Medicine 36 (4) 495-507 (See also: Langer A, Farnot U, Garcia C, Barros F, Victora C, Belizan J, Villar J 1996 The Latin American trial of psychosocial support during pregnancy: effects on mother’s wellbeing and satisfaction. Social Science and Medicine 42 (11) 1589-1597)

Lutenbacher M, Gabbe PT, Karp SM, Dietrich MS, Narrigan D, Carpenter L, Walsh W 2014 Does additional prenatal care in the home improve birth outcomes for women with a prior preterm delivery? A randomized clinical trial. Maternal and Child Health Journal 18: 1142-1154

Mueller-Heubach E, Reddick D, Barnett B, Bente R 1989 Preterm birth prevention: evaluation of a prospective controlled randomized trial. American Journal of Obstetrics and Gynecology 160: 1172-1178

Muender MM, Moore ML, Chen GJ, Seick MA 2000 Cost-benefit of a nursing home telephone intervention to reduce preterm and low-birthweight births in an African American clinic population. Preventative Medicine 30: 271-276

Norbeck JS, Dejoseph JF, Smith RT 1996 A randomized trial of an empirically-derived social support intervention to prevent low birthweight among Africian American women. Social Science & Medicine 43:947-954

Oakley A, Rajan L, Grant A 1990 Social support and pregnancy outcome. British Journal of Obstetrics & Gynaecology 97: 155-162

Ross MG, Sandhu M, Bemis R, Nessim S, Bragonier JR, Hobel C 1994 The west Los Angeles preterm birth prevention project: II. Cost-effectiveness analysis of high-risk pregnancy interventions. Obstetrics & Gynaecology 83: 506-511

**T6-3 Interventions for women who smoke - Targeted ‘higher risk’-based model**

Bullock L, Everett KD, Mullen PD, Geden E, Longo DR, Madsen R 2009 Baby BEEP: A randomized controlled trial of nurses' individualized social support for poor rural pregnant smokers. *Maternal & Child Health Journal,* 13**,** 395-406

O’Connor AM, Davies BL, Dulberg CS, Buhler PL, Nadon C, McBride BH, Benzie RJ 1992 Effectiveness of a pregnancy smoking cessation program. *JOGNN - Journal of Obstetric, Gynecologic, & Neonatal Nursing,* 21**,** 385-92

Ondersma SJ, Svikis DS, Lam PK, Connors-Burghe VS, Ledgerwood DM, Hopper JA 2012 A randomized trial of computer-delivered brief intervention and low-intensity contingency management for smoking during pregnancy. *Nicotine & Tobacco Research,* 14**,** 351-60

Parker DR, Windsor RA, Roberts MB, Hecht J, Hardy NV, Strolla LO, Lasater TM 2007 Feasibility, cost, and cost-effectiveness of a telephone-based motivational intervention for underserved pregnant smokers. *Nicotine & Tobacco Research,* 9**,** 1043-1051

Pbert L, Ockene JK, Zapka J, Ma YS, Goins KV, Oncken C, Stoddard AM 2004 A community health center smoking-cessation intervention for pregnant and postpartum women. *American Journal of Preventive Medicine,* 26**,** 377-385

Petersen L, Handel J, Kotch J, Podedworny T, Rosen A 1992 Smoking reduction during pregnancy by a program of self-help and clinical support. *Obstetrics and Gynecology,* 79**,** 924-930

Ruger JP, Emmons KM, Kearney MH, Weinstein MC (2009) Measuring the costs of outreach motivational interviewing for smoking cessation and relapse prevention among low-income pregnant women. *BMC Pregnancy & Childbirth,* 9**,** 46

Secker-Walker RH, Solomon LJ, Flynn BS, Skelly JM, Mead PB 1998 Reducing smoking during pregnancy and postpartum: physician's advice supported by individual counseling. *Preventive Medicine,* 27**,** 422-30 (See also: Secker-Walker RH, Solomon LJ, Flynn BS, Skelly JM, Mead PB 1998 Smoking relapse prevention during pregnancy. A trial of coordinated advice from physicians and individual counseling. *American Journal of Preventive Medicine,* 15**,** 25-31)

Tappin DM, Lumsden MA, McKay C, McIntyre D, Gilmour H, Webber R, Cowan S, Crawford F, Currie F 2000 The effect of home-based motivational interviewing on the smoking behaviour of pregnant women: a pilot randomized controlled efficacy study. *Ambulatory Child Health,* 6**,** 34-35

**T6-4 Interventions for women with anxiety or mental health issues - Targeted ‘higher risk’-based model**

Bastani F, Hidarnia A, Montgomery KS, Aguilar-Vafael ME, Kazemnejad A 2006 Does relaxation education in anxious primigravid Iranian women influence adverse pregnancy outcomes? A randomized controlled trial. Journal of Perinatal and Neonatal Nursing 20: 138-146

Brugha T, Wheatley S, Taub N, Culverwell A, Friedman T, Kirwan P, Jones D, Shapiro D 2000 Pragmatic randomized trial of antenatal intervention to prevent post-natal depression by reducing psychosocial risk factors. Psychological Medicine 30: 1273-1281

Guardino CM, Dunkel Schetter C, Bower JE, Lu MC, Smalley SL 2014 Randomised controlled pilot trial of mindfulness training for stress reduction during pregnancy. *Psychology & Health,* 29**,** 334-49

Ortiz Collado MA, Saez M, Favrod J, Hatem M 2014 Antenatal psychosomatic programming to reduce postpartum depression risk and improve childbirth outcomes: a randomised controlled trial in Spain and France. BMC Pregnancy and Childbirth 14: 22-34

Petrou S, Cooper P, Murray L, Davidson LL 2006 Cost-effectiveness of a preventative counselling and support package for postnatal depression. International Journal of Technology Assessment in Health Care 22: 443-453

Rahman A, Malik A, Sikander S, Roberts C, Creed F 2008 Cognitive behaviour therapy-based intervention by community health workers for mothers with depression and their infants in rural Pakistan: a cluster-randomised controlled trial. Lancet 372: 902-909

Richter J, Bittner A, Petrowski K, Junge-Hoffmeister J, Bergmann S, Joraschky P, Weidner K 2012 Effects of an early intervention on perceived stress and diurnal cortisol in pregnant women with elevated stress, anxiety, and depressive symptomatology. Journal of Psychosomatic Obstetrics and Gynecology 33 (4) 162-170

Saisto T, Samela-Aro K, Nurmi JE, Kononen T, Halmesmaki E 2001 A randomized controlled trial of intervention in fear of childbirth. Obstetrics and Gynecology 98: 820-826

Webster J, Linnane J, Roberts J, Starrenburg S, Hinson J, Dibley L 2003 IDentify, Educate and Alert (IDEA) trial: an intervention to reduce postnatal depression. British Journal of Obstetrics and Gynaecology 10: 842-846

**T6-5 Interventions for overweight/obese women and/or women at risk of gestational diabetes mellitus - Targeted ‘higher risk’-based model**

Harrison CL, Lombard CB, Strauss BJ, Teede HJ 2013 Optimizing healthy gestational weight gain in women at high risk of gestational diabetes: a randomized controlled trial. *Obesity,* 21**,** 904-9.

Oostdam N, Bosmans J, Wouters MGAJ, Eekhoff EMW, van Mechelen W, van Poppel MNM 2012 Cost-effectiveness of an exercise program during pregnancy to prevent gestational diabetes: results of an economic evaluation alongside a randomised controlled trial. *BMC Pregnancy & Childbirth,* 12**,** 64

Perez Ferre N, Galindo M, Fernandex MD, Velasco V, Runkle I, de la Cruz MJ, Martin Rojas-Marcos P, Del Valle L, Calle-Pascual AL 2010 The outcomes of gestational diabetes mellitus after a telecare approach are not inferior to traditional outpatient clinic visits. *International Journal of Endocrinology Print,* 2010**,** 386941

Poston L, Briley AL, Barr S, bell R, Coker H, Coxon K, Essex HN, Hunt C, Hayes L, Howard LM, Khazaezadeh N, Kinnuen T, Nelson SM, Oteng-Ntim E, Robson SC, Sattar N, Seed PT, Wardle J, Sanders TAB, Sandall J 2013 Developing a complex intervention for diet and activity behaviour change in obese pregnant women (the UPBEAT trial); Assessment of behavioural change and process evaluation in a pilot randomised controlled trial. *BMC Pregnancy and Childbirth,* 13

Quinlivan JA, Lam LT, Fisher J 2011 A randomised trial of a four-step multidisciplinary approach to the antenatal care of obese pregnant women. *Australian & New Zealand Journal of Obstetrics & Gynaecology,* 51**,** 141-6.

**T6-6 Other ‘higher risk’ clinical / psychosocial target groups - Targeted ‘higher risk’-based model**

Barlow J, Davis H, McIntosh E, Jarrett P, Mockford C, Stewart-Brown S 2007 Role of home visiting in improving parenting and health in families at risk of abuse and neglect: results of a multicentre randomised controlled trial and economic evaluation. Archives of Disease in Childhood 92: 229-233

Fraser W, Maunsell E, Hodnett E, Moutquin JM 1997 Randomized controlled trial of a prenatal vaginal birth after cesarean section education and support program. Childbirth Alternatives Post-Cesarean Study Group. *American Journal of Obstetrics & Gynecology,* 176**,** 419-25

McIntosh E, Barlow J, Davis H, Stewart-Brown S 2009 Economic evaluation of an intensive home visiting programme for vulnerable families: a cost-effectiveness analysis of a public health intervention. Journal of Public Health, 31**,** 423-33

O’Connor MJ, Whaley SE 2007 Brief intervention for alcohol use by pregnant women. *American Journal of Public Health,* 97**,** 252-8

Osterman RL, Dyehouse J 2012 Effects of a motivational interviewing intervention to decrease prenatal alcohol use. *Western Journal of Nursing Research,* 34**,** 434-54

Ross R, Sawatphanit W, Suwansujarid T, Stidham AW, Drew BL, Cresswell JW 2013 The effect of telephone support on depressive symptoms among HIV-infected pregnant women in Thailand: an embedded mixed methods study. *Journal of the Association of Nurses in AIDS Care,* 24**,** e13-24.

Sen D 2006 Midwife led intervention during pregnancy improves psychological morbidity in women with twin infants during the first year after birth - a randomised controlled trial. *Journal of Reproductive and Infant Psychology,* 24**,** 276-277 (See also: Carrick-Sen [Carrick**-Sen** DM](javascript:__doLinkPostBack('','ss~~AU%20%22Carrick-Sen%20DM%22%7C%7Csl~~rl','');), [Steen N](javascript:__doLinkPostBack('','ss~~AU%20%22Steen%20N%22%7C%7Csl~~rl','');), [**Robson** SC](javascript:__doLinkPostBack('','ss~~AU%20%22Robson%20SC%22%7C%7Csl~~rl','');). Twin parenthood: the midwife's role--a randomised controlled trial. [BJOG: An International Journal Of Obstetrics And Gynaecology](javascript:__doLinkPostBack('','mdb~~cmedm%7C%7Cjdb~~cmedmjnh%7C%7Css~~JN%20%22BJOG%3A%20an%20international%20journal%20of%20obstetrics%20and%20gynaecology%20%5BBJOG%5D%20NLMUID%3A%20100935741%22%7C%7Csl~~jh','');) [BJOG] 2014 Sep; Vol. 121 (10), pp. 1302-10)

Taft AJ, Small R, Hegarty KL, Watson LF, Gold L, Lumley JA 2011 Mothers' Advocates In the Community (MOSAIC)--non-professional mentor support to reduce intimate partner violence and depression in mothers: a cluster randomised trial in primary care. *BMC Public Health,* 11**,** 178

Turnbull C, Osborn-David A 2012 *Home visits during pregnancy and after birth for women with an alcohol or drug problem*. The Cochrane library. DOI: 10.1002/14651858.CD004456.pub3

**T6-7 ‘Higher risk’ socio-demographic target groups - Targeted ‘higher risk’-based model**

Aracena M, Krause M, Perez C, Mendez MJ, Salvatierra L, Sotto M, Pantoja T, Navarro S, Salinas A, Farah C, Altimir C 2009 A cost-effectiveness evaluation of a home visit program for adolescent mothers. Journal of Health Psychology 14: 878-887

Barlow A, Varipatis-Baker E, Speakman K, Ginsburg G, Friberg I, Goklish N, Cowboy B, Fields P, Hastings R, Pan W, Reid R, Santosham M, Walkup J 2006 Home-visiting intervention to improve child care among American Indian adolescent mothers: a randomized trial. Archives of Pediatrics & Adolescent Medicine 160: 1101-1107

Barlow A, Mullany B, Neault N, Compton S, Carter A, Hastings R, Billy T, Coho-Mescal V, Lorenzo S, Walkup JT 2013 Effect of a paraprofessional home-visiting intervention on American Indian teen mothers’ and infants’ behavioural risks: a randomized controlled trial. American Journal of Psychiatry 170: 83-93

Barlow J, Davis H, McIntosh E, Jarrett P, Mockford C, Stewart-Brown S 2007 Role of home visiting in improving parenting and health in families at risk of abuse and neglect: results of a multicentre randomised controlled trial and economic evaluation. Archives of Disease in Childhood 92: 229-233)

Barlow J, Smailagic N, Bennet C, Huband N, Jones H, Coren E 2011 Individual and group based parenting programmes for improving psychosocial outcomes for teenage parents and their children. Cochrane Database of Systematic Reviews

Barnet B, Duggan AK, Devoe M, Burrell 2002 The effect of volunteer home visitation for adolescent mothers on parenting and mental health outcomes: a randomized trial. Archives of Pediatrics & Adolescent Medicine 156: 1216-1222

Barnet B, Lui J, Devoe M, Alperovitz-Bichell K, Duggan AK 2007 Home visiting for adolescent mothers: effects on parenting, maternal life course, and primary care linkage. Annals of Family Medicine 5: 224-232

Edwards RC, Thullen MJ, Korfmacher J, Lantos JD, Henson LG, Hans SL 2013 breastfeeding and complementary food: randomized trial of community doula home visiting. Pediatrics 132 (S2) 160-166

Fausett M, Gill B, Esplin M, Shields A, Staat B 2014 Centering Pregnancy is associated with fewer early, but not overall, preterm deliveries. *American Journal of Obstetrics and Gynecology,* 1)**,** S9

Ford K, Weglicki L, Kershaw T, Schram C, Hoyer PJ, Jacobson ML 2002 Effects of a prenatal care intervention for adolescent mothers on birth weight, repeat pregnancy, and educational outcomes at one year postpartum. Journal of Perinatal Education 11: 35-38

Ickovics JR, Kershaw TS, Westdahl C, Magriples U, Massey Z, Reynolds H, Rising SS 2007 Group prenatal care and perinatal outcomes – a randomized controlled trial. Obstetrics and Gynecology 110: 330-339 (See also: Ickovics JR, Reed E, Magriples U, Westdale C, Massey Z, Reynolds H, Schindler Rising S, Kershaw TS 2011 Effects of group prenatal care on psychosocial risk in pregnancy: results from a randomised controlled trial. Psychology & Health 26: 235-250 – and - Westdahl C, Kershaw T, Schindler Rising S, Ickovics JR 2008 Group prenatal care improves breastfeeding initiation and duration: results from a two-site randomised controlled trial. Journal of Human Lactation 24 (1) 96)

Kennedy HP, Farrell T, Paden R, Hill S, Jolivet RR, Cooper BA, Rising SS 2011 A randomized clinical trial of group prenatal care in two military settings. Military Medicine, 176, 1169-77.

McLachlan FJ, Altemeier WA, Christensen MJ, Sherrod KB, Dietrich MS, Stern DT 1992 Randomized trial of comprehensive prenatal care for low-income women: effect on infant birth weight. Pediatrics 89: 128-132

Olds DL, Henderson CR, Tatelbaum R, Chamberlin R 1986 Improving the delivery of prenatal care and outcomes of pregnancy – a randomized controlled trial of nurse home visitation. *Pediatrics,* 77**,** 16-28 (See also: Olds DL, Henderson CR, Kitzman H, Eckenrode J, Cole R, Tatelbaum R, 1998 The promise of home visitation: Results of two randomized trials. *Journal of Community Psychology,* 26**,** 5-21 – and - Olds DL 2002 Prenatal and infancy home visiting by nurses: from randomized trials to community replication. *Prevention Science,* 3**,** 153-72 – and - Olds DL, Henderson CR, Phelps C, Kitzman H, Hanks C 1993 Effect of prenatal and infancy nurse home visitation on government spending. *Medical Care,* 31**,** 155-74 – and - Olds DL, Robinson J, O'Brien R, Luckey DW, Pettit LM, Henderson CR, Ng RK, Sheff KL, Korfmacher J, Hiatt S, Talmi A 2002 Home visiting by paraprofessionals and by nurses: a randomized, controlled trial. *Pediatrics,* 110**,** 486-96)

Polley BA, Wing RR, Sims CJ 2002 Randomized controlled trial to prevent excessive weight gain in pregnant women. International Journal of Obesity 26: 1494-1502

Roman LA, Gardiner JC, Lindsay JK, Moore JS, Luo Z, Baer, LJ, Goddeeris JH, Shoemaker AL, Barton LR, Fitzgerald HE, Paneth N 2009 Alleviating perinatal depressive symptoms and stress: a nurse-community health worker randomized trial. Archives of Women’s Mental Health 12: 379-391 (See also: Roman LA, Lindsay JK, Moore JS, Duthie PA, Peck C, Barton LR, Gebben MR, Baer LJ 2007 Addressing mental health and stress in Medicaid-insured pregnant women using a nurse-community health worker home visiting team. Public Health Nursing 24: 239-248

Weis KL, Ryan TW 2012 Mentors offering maternal support: a support intervention for military mothers. JOGNN - Journal of Obstetric, Gynecologic, & Neonatal Nursing, 41**,** 303-14

Wen LM, Baur LA, Simpson JM, Rissel C, Flood VM 2011 Effectiveness of an early intervention on infant feeding practices and “Tummy Time”: a randomized controlled trial. Archives of Pediatric Adolescent Medicine 165 (8) 701-707
